# Efficacy of Surgery Combined with Autologous Bone Marrow Stromal Cell Transplantation for Treatment of Intracerebral Hemorrhage

**DOI:** 10.1155/2015/318269

**Published:** 2015-07-09

**Authors:** Jianxin Zhu, Yilei Xiao, Zhongmin Li, Fabin Han, Taiwu Xiao, Zhiti Zhang, Fengyang Geng

**Affiliations:** ^1^Department of Neurosurgery, Liaocheng People's Hospital of Taishan Medical University, Liaocheng 252000, China; ^2^Neurological Laboratory, Liaocheng People's Hospital of Taishan Medical University, Liaocheng 252000, China; ^3^Department of Hematology, Liaocheng People's Hospital of Taishan Medical University, Liaocheng 252000, China

## Abstract

Bone marrow stromal cells (BMSCs) may differentiate into nerve cells under a certain condition; however, the clinical application for treating nervous system disease remains unclear. The aim is to assess the safety profile, feasibility, and effectiveness of surgery combined with autologous BMSCs transplantation for treating ICH. 206 ICH patients who had received surgical procedure were divided into transplantation (*n* = 110) or control group (*n* = 96). For transplantation group, BMSCs were injected into the perihemorrhage area in the base ganglia through an intracranial drainage tube 5.5 (3.01–6.89) days after surgery, followed by a second injection into the subarachnoid space through lumbar puncture 4 weeks later. Neurologic impairment and daily activities were assessed with National Institute Stroke Scale (NIHSS), Barthel index, and Rankin scale before transplantation and 6 months and 12 months after transplantation. Our results revealed that, compared with control group, NIHSS score and Rankin scale were both significantly decreased but Barthel index was increased in transplantation group after 6 months. Interestingly, no significant difference was observed between 12 months and 6 months. No transplantation-related adverse effects were investigated during follow-up assessments. Our findings suggest that surgery combined with autologous BMSCs transplantation is safe for treatment of ICH, providing short-term therapeutic benefits.

## 1. Introduction

ICH ranks the third leading cause of death, following cardiovascular disease and malignant tumors. Most cases with ICH have various neurological deficits, including aphasia, hemiparalysis, and sphincter abnormalities [1]. Repair of damaged nerve tissue and recovery of neurological function are unsatisfactory with traditional approaches such as surgery, physical rehabilitation, medications, and hyperbaric oxygen therapy [2].

Stem cell transplantation and cell engineering have become candidates for treatment of various nerve injuries [3]. Currently, the primary sources of adult stem cells for therapeutic purposes are bone marrow, umbilical cord blood, and adipose and brain tissues of adult animals. Allogeneic stem cell therapy has been seriously constrained because of difficulty of obtaining, and ethical and legal restrictions have also limited access to material. Bone marrow stem cells, including hematopoietic stem cells and bone marrow stromal cells (BMSCs), are pluripotent and can self-renew. Thus, bone marrow stromal cells (BMSCs) have drawn increasing attention as a rich resource that has multipotential differentiation and is convenient to obtain [4, 5]. Limited information is available on treating ICH with autologous BMSCs, and its effectiveness, safety, time course, and methods have not been established.

A group of ICH patients were treated with autologous BMSCs transplantation after surgery in the Department of Neurosurgery, Liaocheng People's Hospital of Taishan Medical University, China, based on the previous results [6]. The treatment was safe and effective, based on the results of comparison with controls during evaluations conducted 6 and 12 months following transplantation.

## 2. Materials and Methods

### 2.1. General Information

#### 2.1.1. Clinical Information

Transplant procedures were approved by the Ethics Committee of Liaocheng People's Hospital (approval ID: 20080008). From January 2009 to September 2011, 755 cases with acute ICH were enrolled in the Department of Neurosurgery. 540 cases were excluded for not meeting the criteria (*n* = 431) or refused to participate in this study (*n* = 109). 215 ICH patients were enrolled in this study. Informed consent was obtained from all participants or their authorized clients.

#### 2.1.2. Inclusion and Exclusion Criteria

The inclusion criteria used for the study were (1) <80 years old; (2) Glasgow Coma Scale score of 5–12; (3) ICH location on CT scan being limited in the brain basal ganglia; (4) indications for decompression surgery: ICH volume > 20 mL on CT scan and > 10 mm shift of brain from the midline.

The exclusion criteria used for the study were (1) ICH caused by factors such as head injuries, anticoagulants, or tumor, excluding hypertension; (2) a history of allergy; (3) mild ICH focal neurological deficits with no indication for decompressive craniotomy or surgical evacuation of hematoma; (4) concurrent chronic illnesses such as hepatic or renal dysfunction; (5) coagulation disorders; and (6) body temperature being more than 37.5 degrees before transplantation.

After surgical drainage and decompressive craniotomy, the selected patients or their relatives were further consulted on the potential benefits and risks of this BMSCs treatment, particularly on the uncertainties in its clinical effects and long-term side effects. In the end, full written consent on the cell implantation was obtained from 114 patients. The other 101 patients who did not wish to receive BMSCs treatment participated in this study as the control group.

### 2.2. Treatment Procedures

#### 2.2.1. Surgical Treatment

After preoperative examinations, detailed surgery plans were designed for the individuals based on their clinical information and hematoma volume. Indwelling drainage tubes were maintained in the cavities of cases receiving hematoma evacuation via small bone window craniotomies or bone flap craniotomies.

#### 2.2.2. Autologous BMSC Preparation

Bone marrow aspiration was operated by a hematologist under local anesthesia in a class 100 laminar flow operating room, 5.5 (3.01–6.89) days after surgery. Autologous bone marrow (200 mL) was collected with sodium citrate to prevent clotting and added in stem cell isolating reagent (Wealthlin Science & Technology Inc., Canada) on a superclean worktable. The cells were centrifuged at 1100 g for 30 minutes. The cells in the interphase were recuperated and washed twice with PBS (300 g for 10 minutes). A stem cell suspension (0.5 mL) was obtained after removing red cells, centrifuging, harvesting, and flushing.

#### 2.2.3. The First Autologous BMSC Implantation

The harvested cell suspension in a volume of 0.25 mL was diluted 1 : 20 with saline. An aliquot (0.5%) was removed at 4°C in the neurological laboratory to count nucleated cells and BMSCs, with the rest being used for transplantation. Stem cells were administered through indwelling drainage tubes, which had been drawn to the rim of the hematoma cavity before injection and were removed after injection.

#### 2.2.4. Autologous BMSC Culture

**Figure 1:**
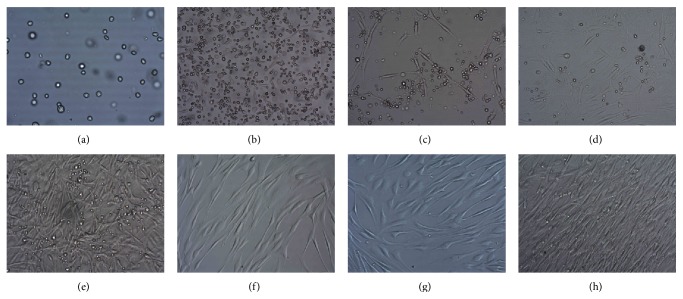
*In vitro* culture of autologous bone marrow mesenchymal stem cells. (a) Day 1 × 380 no staining; (b) Day 3 × 380 no staining; (c) Day 5 × 380 no staining; (d) Day 10 × 380 no staining; (e) Day 14 × 380 no staining; (f) Day 18 × 380 no staining; (g) Day 21 × 380 no staining; and (h) Day 26 × 380 no staining.

The stromal cell suspension in a volume of 0.25 mL was sent for subculture using the following procedures: harvested stem cells were suspended in *α*-MEM (Invitrogen, Carlsbad, CA, USA, Cat: 12714010S) (2–7.5 × 10^6^ cells/mL) at 10% of fetal bovine serum (FBS, GIBCO, Carlsbad, CA, USA, Cat: 16000-004) and inoculated (2.5–10 × 10^5^ cells/cm^2^); medium was changed 2-3 d later to remove nonadherent cells, followed by a subsequent change 3–5 d later. Digestion passage was performed when confluence attained 80%. Microbial levels, cell counts, and survival rates were independently examined by a third party to ensure culture quality. Cells were harvested and counted at passage 3 (Figure 1).

#### 2.2.5. The Second Autologous BMSC Implantation

Under local anesthesia, BMSCs suspensions were injected into the subarachnoid space via a lumbar puncture. An interval of 4 wks between transplants constituted a treatment cycle.

### 2.3. Cell Counts

Nucleated cells in collected samples and cell suspensions were counted with microscopy, and BMSCs were counted using flow cytometry. CD29+, CD44+, CD106+, and CD166+ cells were recognized with corresponding antibodies. Briefly, the cells were collected and incubated with the following monoclonal antibodies for 1 hour at room temperature: CD106-PE (Cat: 555647), CD29-FITC (Cat: 555005), CD44-PE (Cat: 550989), and CD166-PE (Cat: 559263). All antibodies were purchased from Becton Dickinson (San Jose, CA). Then cells were washed 3 times with phosphate buffered saline (PBS). Cells were measured by FACSCalibur (BD Biosciences).

### 2.4. Functional Assessment

Six months after BMSCs implantation, the patient's swallowing difficulty (Kubota water experiment), muscle strength (MMT), muscle tension (MAS), language ability (Part “V” of GCS), calculation (Part “Calculation” of MoCA), cognition (MoCA), and responsiveness to painful stimulation (Part “M” of GCS) were separately evaluated by a neurologist who was blinded to patient's treatment according to the international uniform scale.

**Figure 2:**
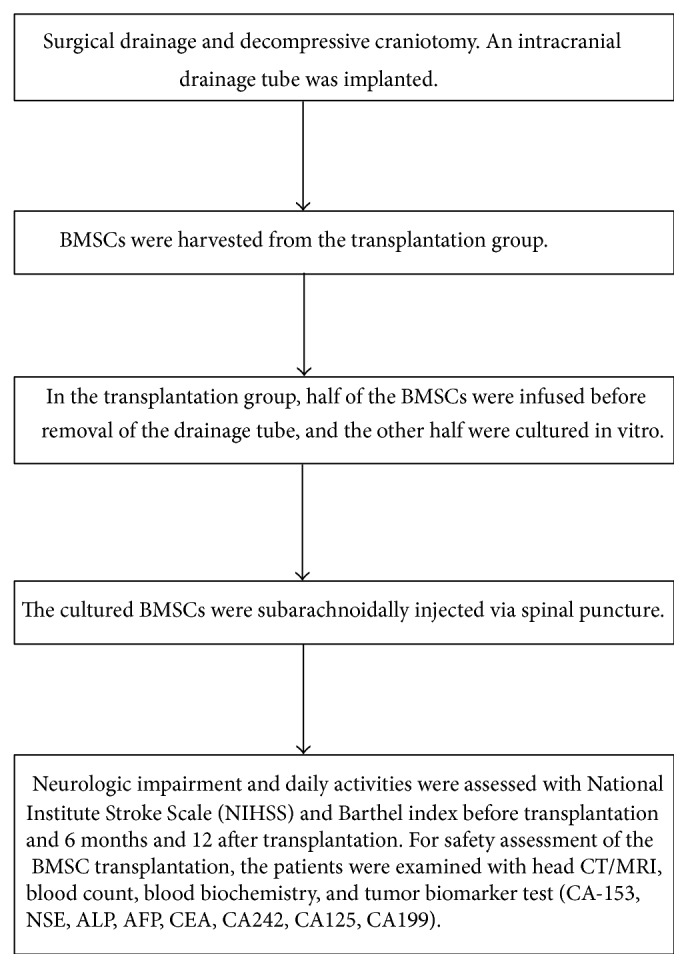
Flow diagram of the procedures of the surgery and the monitoring process of the safety and efficacy of BMSCs.

NIHSS score, Barthel index, and Rankin scale were assessed before transplantation and 6 and 12 months after transplantation. Statistical comparisons were performed on results of the transplantation group obtained before and after transplantation and between the transplantation and control groups. The safety of autologous BMSCs transplantation was monitored using cranial CT or MRI, blood routine examination, biochemical indices, and tumor biomarkers (including CA-153, NSE, ALP, AFP, CEA, CA242, CA125, and CA199) before transplantation and at 6 and 12 months after the transplantation.  Figure 2 displayed the flow diagram of the procedures of the surgery and the monitoring process of the safety and efficacy of BMSCs.

### 2.5. Statistical Analysis

Data were presented as the mean ± SEM or median (interquartile range). SPSS software package (version 13.0; SPSS, Chicago, IL, USA) was used for the statistical analysis. The distribution of the samples was determined by Kolmogorov-Smirnov test. The data from experiments were analyzed by Student's* t*-test or nonparametric Mann-Whitney test, and *P* < 0.05 was considered as statistical significance.

## 3. Results

### 3.1. General Findings

Four patients from the implantation group and five from the control did not return to the hospital for schedule follow-up, who were excluded from the final analysis. 110 patients in the implantation group and 96 patients in the control group were included in the final analysis. There was no significant difference in the demographic data including age, sex, neurological findings, and the mean volumes of bleeding between the two groups (*P* > 0.05, Table 1). Simple drainage, evacuation of hematoma, or decompressive craniotomy was performed in all patients, with no significant difference in the surgical methods between the two groups (*P* > 0.05, Table 2).

### 3.2. Cell Counting

**Figure 3:**
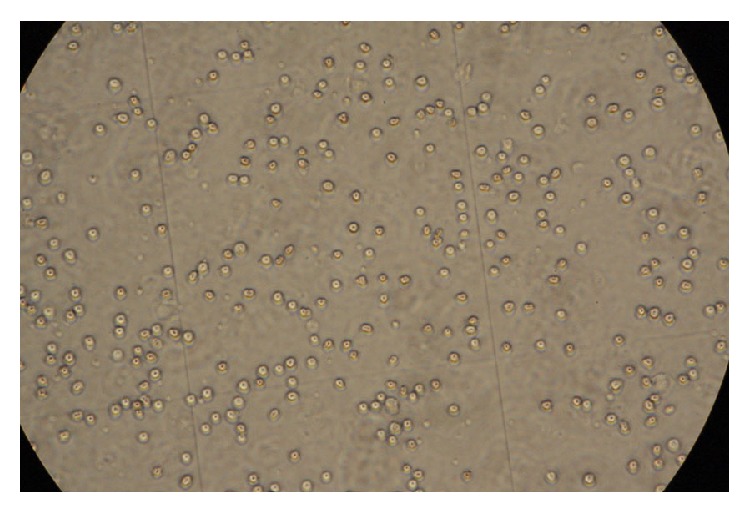
Nucleated cell counts. Monocytes were observed to be numerous, uniform in morphology, and with round cell bodies (×40).

Samples were obtained from 206 cases and sent to the Neurological Laboratory for Nucleated Cell and BMSC Counts. Monocytes were uniform in morphology with a round body, although a few polykaryocytes were detected (Figure 3). Nucleated cell, isolated BMSC, and cultured BMSC counts were summarized (Table 3).

### 3.3. Functional Assessment

The proportion of patients who experienced complete recovery from pretreatment swallowing difficulties, reduced muscle strength or tension, compromised language and cognition, or reduced responses to painful stimulation are listed in Table 4. A higher rate of complete recovery in the above indices was found in the transplantation group (Table 4). Improvement in one or more of the above neurological and functional assessment measures was observed in 102 (92.7%) transplantation group patients and in 42 control group patients (43.8%, *P* = 0.0027). There were 12 cases and 3 cases of complete recovery of all symptoms in MSC group and control group, respectively (Table 4).

NIHSS, Barthel's scores, and Rankin scale were obtained before transplantation and 6 and 12 months after transplantation for the two groups. All variables are confirmed to be not normally distributed. The two group's NIHSS scores and Rankin scale were reduced and Barthel's scores were increased 6 months after transplantation. NIHSS scores and Rankin scale in the transplantation group were lower, whereas Barthel scores were higher than those in the control group (*P* < 0.01). No significant difference in the scores was observed between 12 months and 6 months (Table 5).

### 3.4. Safety of Transplantation

**Figure 4:**
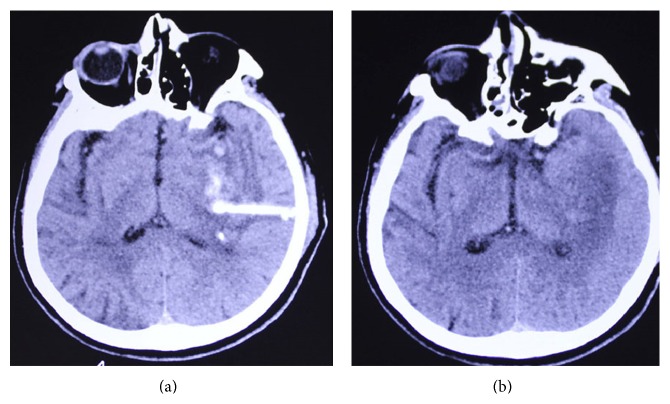
Cranial CT images of a patient before (a) and 6 months after BMSCs transplantation (b).

Seven patients (6.36%) from the transplantation group were identified with low grade fever (<38.5°C) in 24 h after the first transplantation. Two patients (1.81%) also experienced low grade fever after the second transplantation. The fever in all 9 patients subsided in 3 days without specific pharmacological intervention. Except for one patient (0.91%) in the transplantation group with an increase in CA-153, no abnormalities in brain CT or MRIs (Figure 4), routine and biochemical blood indices, or cancer biomarkers (CA-153, NSE, ALP, AFP, CEA, CA242, CA125, and CA199) were identified during follow-up studies. The patient was diagnosed with lung cancer later. No transplantation-related adverse effects were investigated during follow-up assessment.

## 4. Discussion

Bone marrow stromal cells (BMSCs) used in our study have significant advantages: (1) ease of obtaining large numbers of cells with bone marrow aspiration; (2) fast amplification in a short period; (3) the probability of these cells crossing the blood brain barrier [7]; and (4) immune rejection not occurring with autologous transplantation. Like stem cells, BMSCs have the potency of multidirectional differentiation. Their potential to differentiate into neuronal cells is the basis for treatment of neurovascular diseases [8]. In a recent study where BMSCs were transplanted from male to female rats with ICH BrdU positive cells were significantly increased, based on immunoassays for BrdU and neuromarkers [9]. Mahmood et al. [10] and Lu et al. [11] demonstrated that BMSCs could migrate to the sites of brain damages, differentiate into glial cells, and express neuronal marker and the functions of the damaged neurons were found improved. Zhang et al. [12] also demonstrated that the levels of the nerve growth factors increased significantly, which could promote BMSCs' differentiation into nervous tissue.

The safety of the transplantation is our utmost concern. In a study performed by Rice et al. [13], autologous BMSCs transplantation was proved to be safe in treating relapsing-progressive multiple sclerosis. In the present study, low grade fever (<38.5°C) was observed in 9 patients (8.18%) from the transplantation group within the first 24 hours of transplantation. Since the transplanted BMSCs were from autologous bone marrow, we suggest that this temporary pyrexia may result from endogenous pyrogen release from monocytes and granulocytes or flaws in aseptic procedures but not immune rejection. Several findings reveal a relationship between BMSCs and cancer, providing the hypothesis that cancer is originated from cancer stem cells. Recently, it has been reported that transplanted BMSCs readily differentiate into glial cells under the appropriate host microenvironment. Brain injury and degeneration are always accompanied by strong gliosis, injury to the blood brain barrier, and inflammation. This may trigger glial regeneration signals, weaken neuronal regeneration signals, and thereby bias differentiation of transplanted BMSCs [14]. It is currently unknown whether such gliosis will drive transplanted BMSCs to differentiate into gliosis lesions or glioma. There is no report of brain tumor development following either embryonic brain or BMSCs transplantation. Tumor biomarker and brain CT or MRI abnormalities were not observed in the cases in our study during postsurgical examination for up to three years.

The curative effect of BMSCs transplantation for nerve injuries occurs via multiple pathways. First, transplanted BMSCs migrate towards the lesion site and fuse into tissues, replace the damaged cells, repair and recreate the neural circuitry, and thus repair neurological functions. Second, the interactions between BMSCs and host nerve tissue may generate cytokines that improve neurological function recovery. Sixty ICH cases that receive autologous BMSC transplantation have improved quality of life in a study conducted by Li et al. [6]. In our study, we increased the number of cells to 10^7^ via three generations of culture. As a result, 91.9% (101/110) of transplantation group cases showed improved performance and quality of life 6 months after surgery, characterized by lower NIHSS scores and higher Barthel's scores. Meanwhile, the cases (*n* = 9) without significant improvement were not worse than before. Comparisons between follow-up results obtained 6 and 12 months following transplantation showed no significant differences, even a moderate decrease in Barthel's scores 12 months after transplantation indicating that the effective window of recovery was not prolonged. We speculated that some changes have occurred with time prolongation, such as neural plasticity, functional reorganization, lesions gliosis, and scar formation. BMSCs might not play an important role in this procession. We suggested that BMSCs should be transplanted within 6 months for the best effect.

The location of grafts relative to lesions was suggested by Kelly et al. to be critical for the survival of BMSCs transplanted for treatment of neurovascular diseases [15]. Currently, most BMSCs are transplanted locally via cerebrospinal fluid circulation or venous access. We propose that intracranial injection at the basal ganglia offers the advantages of avoiding crossing the blood brain barrier and direct participation in peri-injection site recovery due to BMSCs clustering, proliferation, and migration around the injection site. The drainage tube was withdrawn to the rim of hematoma cavity before injection in order to avoid the lesion microenvironment, where there were severe cell death, edema, and various inflammatory products that can compromise transplanted cell's survival [16]. In contrast, transplanting cells to sites at the lesion's periphery can improve survival of transplanted cells and surviving neural cells around the lesion (ischemic penumbra), which is the best strategy for significantly reducing the extent and prevalence of disability [17]. If* in situ* transplantation is impossible, injection into the subarachnoid space through a lumbar puncture provides another convenient, low-damage, and repeatable strategy for transplanting stem cells. All 110 cases in the transplantation group received cells via a lumbar puncture to the subarachnoid space for their second transplantation as a means of eliminating the need to repeatedly indwell a tube.

The optimal time for transplanting BMSCs for treatment of ICH remains unclear, although the data from animal and clinical models suggest that BMSCs can survive when administered during a range of pathological stages [18–20]. We speculate that the acute phase following hemorrhage may hinder survival of transplanted cells. A possible explanation is that hypoxia, ischemia, coagulation, and dissolution and absorption of the hematoma trigger the release of bioactive substances, such as excitatory neurotransmitters, free radicals, and inflammatory factors that create a microenvironment that favors development of cell apoptosis [21]. Although some inflammatory factors have been reported to promote cell proliferation and chemotaxis [17], high level of noxious mediators may compromise survival of grafted cells. On the other hand, natural recovery of neurological functions following hemorrhage should be considered so that the optimal timing for repair of nervous structures is not missed by transplants that occur months or years later. Indeed, it has been suggested by Urdzíková et al. that longer delays following injury result in denser glial scars at the lesion site, which harms the growth of neural stem cells and dampens their role in recovery [22]. The transplantation timing in our study was the first chosen to be approximately one week following hemorrhage, when evacuation was finished and the acute phase had nearly ended. This may not be the optimal timing for survival of transplanted stem cells, but transplanting before removal of the drainage tube balances* in situ* transplantation and possible injury due to placement of a second indwelling. We did also perform the clinical evaluations 3 weeks after first infusion, such as NIHSS and Barthel's score, and found that there was no significant difference compared with the control group, although the value was higher than that of control group (data not shown). Therefore, we did the second infusion. Since the BMSCs were acquired with a low yield of 10^5^ cells, we amplified the number of cells to 10^7^ via three cycles. Transplantation of these increased cells into the subarachnoid space about one month after surgery is between the acute phase and the formation of glial scar, and this helps to solidify and improve the curative effect.

BMSCs transplantation provides a new approach for the treatment of neurological dysfunction resulting from ICH. Our result revealed that this study was a success of large scale clinical trial on BMSC transplantation in ICH patients, which is in line with previous study in the therapy of ICH [23, 24]. However, the present study has several limitations such as large sample size, short follow-up period, and lack of direct evidence of neural cell differentiation. Further clinical exploration and follow-up study are needed to determine the optimal site and timing of transplantation, as well as the potential for long-term tumorigenesis. In spite of such shortcomings, the improved clinical outcomes of our cases are encouraging. Autologous BMSC transplantation is a convenient, safe, and effective approach that should improve the recovery of injured neurological functions and increase patient quality of life following ICH. The aims of the present study are to increase the cell purity and identify the best time and best method for the transplantation to help treating more neurological diseases. BMSCs transplantation will benefit the treatment of various neurological diseases with further development of medical techniques as research progresses.

## Figures and Tables

**Table 1:** Demographic data of patients in transplantation and control groups.

	Transplantation (*n* =110)	Control (*n* = 96)	*P*
Mean age (year)	57.2 ± 3.9 (32–75)	58.5 ± 3.0 (36–72)	0.672
Sex (M)	58 (52.7%)	51 (53.1%)	0.821
Unconsciousness	60 (54.5%)	70 (72.9%)	0.089
Loss of language	40 (36.4%)	40 (41.7%)	0.684
Loss of mobility	104 (94.5%)	84 (87.5%)	0.412
Affected limb muscle strength			
Grade 0	40 (36.4%)	42 (43.8%)	0.583
Grades I-II	38 (34.5%)	33 (28.1%)	0.763
Grades III–IV	26 (23.6%)	15 (15.6%)	0.674
Bleeding volume, based on CT scan			
20–30 mL	32 (29.1%)	45 (46.9%)	0.341
30–50 mL	58 (52.7%)	31 (32.3%)	0.185
>50 mL	20 (18.2%)	20 (20.8%)	0.554
Glasgow Coma Scale score (median (interquartile range))	9 (4.56–12.91)	10 (4.89–13.11)	0.147

**Table 2:** Comparison of surgical management.

	Transplantation (*n* = 110)	Control (*n* = 96)	*P*
Time form hemorrhage onset to surgery (h)	5.2 ± 1.2	5.5 ± 1.5	0.225
Simple drainage	47 (42.7%)	31 (32.3%)	0.177
Hematoma evacuation through small skull window	52 (47.3%)	50 (52.1%)	0.558
Hematoma evacuation through craniotomy	11 (10%)	15 (15.67%)	0.628

Numbers are expressed as mean ± SD.

**Table 3:** Transplantation group cell counts (total number achieved).

	Maximum count	Minimum count	Mean
Nucleated cells	5.85 × 10^9^	6.89 × 10^8^	(4.01 ± 1.52) × 10^9^
Mesenchymal cells after isolation	1.67 × 10^6^	8.24 × 10^5^	(9.67 ± 3.89) × 10^5^
Mesenchymal cells after culture	1.28 × 10^8^	6.87 × 10^7^	(8.47 ± 3.54) × 10^7^

**Table 4:** Complete recovery of neurological functions 6 months after surgery in patients who had significant impairment after intracerebral hemorrhage.

	Transplantation	Control	*P*
Swallowing	27/56 (48.2%)	6/47 (12.8%)	0.019
Muscle tension	23/70 (32.9%)	6/62 (9.7%)	0.008
Muscle strength	41/104 (39.4%)	11/84 (13.1%)	0.015
Language	19/40 (47.5%)	9/40 (22.5%)	0.032
Calculation	22/55 (40%)	6/48 (12.5%)	0.007
Cognition	21/68 (30.9%)	6/47 (12.8%)	0.025
Response to painful stimulation	25/60 (41.7%)	8/70 (11.4%)	0.034
Complete recovery of all symptoms	12/110 (10.9%)	3/96 (3.12%)	0.017

**Table 5:** NIHSS, Barthel's scores, and Rankin scale for transplant and control groups before surgery and 6 and 12 months after surgery.

	Transplantation	Control	*P*
	(*n* = 110)	(*n* = 96)	
NIHSS			
Baseline	21 (10.28–29.72)	22 (9.68–27.32)	0.675
After 6 months	10.5 (4.25–16.75)	14.5 (7.58–20.42)	0.009
After 12 months	9 (4.11–15.64)	13.5 (7.25–19.75)	0.002
Barthel's score			
Baseline	27.5 (14.68–39.65)	26 (12.77–46.47)	0.239
After 6 months	62 (31.57–82.43)	41.5 (19.38–63.11)	0.004
After 12 months	69 (37.71–88.26)	49.5 (24.26–69.34)	0.013
Rankin scale			
Baseline	3.5 (1.82–4.66)	3.5 (1.96–4.52)	0.889
After 6 months	2.5 (1.21–3.84)	3 (1.79–4.23)	0.026
After 12 months	2 (1.17–3.36)	2.5 (1.56–3.89)	0.038
